# A novel *FBN1* mutation in a Chinese family with isolated ectopia lentis

**Published:** 2012-04-13

**Authors:** Guoxing Yang, Meifang Chu, Xinling Zhai, Jialiang Zhao

**Affiliations:** 1 Department of Ophthalmology, Peking Union Medical College Hospital, Chinese Academy of Medical Sciences and Peking Union Medical College, Beijing, People’s Republic of China; 2 Department of the Ophthalmology, Xi'an fourth Hospital. Xi'an, Shanxi, People’s Republic of China; 3 Department of the Ophthalmology, 411 Military Hospital of PLA, Shanghai, People’s Republic of China

## Abstract

**Purpose:**

To identify the genetic defect in an autosomal dominant isolated ectopia lentis (EL) family.

**Methods:**

Detailed family history and clinical data were collected from the family including sixteen patients with isolated EL. Blood samples of nine patients, one normal person and two unknown children’s were collected. Genomic DNA was extracted from leukocytes of peripheral blood. Genotyping was performed by microsatellite markers and logarithm-of-odds (LOD) scores were calculated using the LINKAGE Programs. Mutation screening in the candidate gene, fibrillin-1 (*FBN1*), was performed by direct sequencing.

**Results:**

Linkage to the *FBN1* locus is verified. Mutation screening in *FBN1* identified a C>T transition at nucleotide position c.2920. This nucleotide change results in the cysteine substitution for highly conserved arginine at codon 974 (p.R974C). This mutation is identified in all affected individuals but is not found in 50 control healthy people.

**Conclusions:**

A novel mutation of *FBN1* results in an arginine to cysteine residue (p.R974C) substitution, which is responsible for the patients with isolated EL in this Chinese family.

## Introduction

Ectopia lentis (EL) is an inherited connective disorder characterized by lens dislocation. Most cases of EL are associated with Marfan syndrome, a genetic autosomal dominant disorder that is characterized by manifestations involving the cardiovascular, skeletal, and ocular systems [1].

Mutations of the fibrillin-1 (*FBN1*) gene on chromosome 15q have been described in patients with classical Marfan syndrome, neonatal MFS, aortic aneurysms, ectopia lentis, Marfanoid skeletal features, familial arachnodactyly, Shprintzen-Goldberg syndrome, Weill-Marchesani syndrome, and severe progressive kyphoscoliosis [2-6].

An isolated EL pedigree has been reported several times in different races [7-10]. This kind of phenotype may be an independent subtype caused by specific *FBN1* mutations and other regulatory factors.

In this study, we recruited a Chinese family affected with isolated EL. Linkage to the *FBN1* locus is identified. Mutation screening in *FBN1* identified a C>T transition at nucleotide position c.2920. This nucleotide change results in the substitution of highly conserved arginine by cysteine at codon 974 (p.R974C). The mutation is cosegregated in the patients but not found in the 50 control subjects from the same ethnic background.

## Methods

### Patients and clinical data

**Figure 1:**
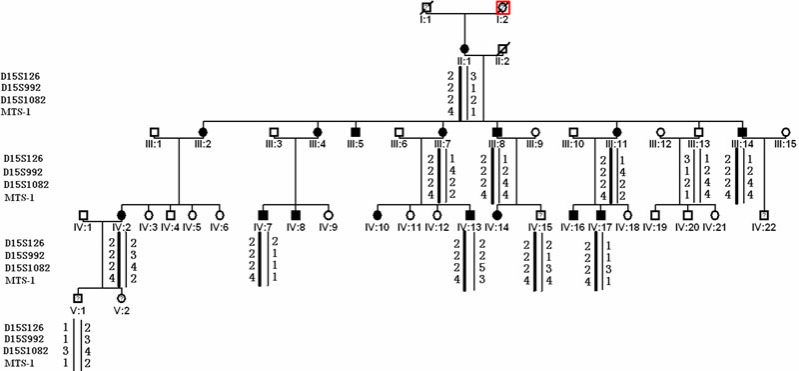
Pedigree and haplotype of the family. A five**-**generation pedigree with twelve available members is shown. Four markers adjacent to *FBN1* were selected. The disease haplotype (represented by the black bar) cosegregated in all affected members but was not shared by unaffected ones.

The five-generation family enrolled in this study was found in a Northwestern province of China. Clinical examination, peripheral blood collection, and DNA extraction were performed in the Department of Ophthalmology, Peking Union Medical College Hospital, Beijing, China. Informed consent in accordance with the Declaration of Helsinki and the Institutional Review Board and Ethics Committee of Peking city was obtained from all participants. The family includes sixteen confirmed patients with isolated EL. Blood samples of nine patients, one normal person, and two younger children (the symptom of ectopia lentis occurs from 17 to 20 in this family) were collected (Figure 1). Clinical data of these subjects was ascertained by case records of surgeries and detailed ocular examinations.

### Genotyping and linkage analysis

With fluorescently labeled microsatellite markers (Sangon Biotech Co,. Ltd, Shanghai, China), linkage analysis of candidate gene region at 15q was done on the DNA samples from 12 ophthalmologically examined individuals. Two-point linkage analysis was performed by MLINK from LINKAGE Program Package.

### Mutation analysis

All coding exons of *FBN1* were amplified by polymerase chain reaction (PCR) using a set of sixty-five pairs of primers (Appendix 1) [11]. The standard PCR reaction (50 μl) contained 80 ng of genomic DNA, 10 pM each primer, 25 μl TaqMix and water. The thermal profile included denaturation for 5 min, followed by 36 cycles of denaturation (30 s at 95 °C), annealing (30 s at 54 °C-60 °C), and extension (45 s at 72 °C), followed by a final step of extension (10 min at 72 °C). The PCR products were sequenced on an ABI3730 Automated Sequencer (PE Biosystems, Foster City, CA).

## Results

### Clinical findings

**Figure 2:**
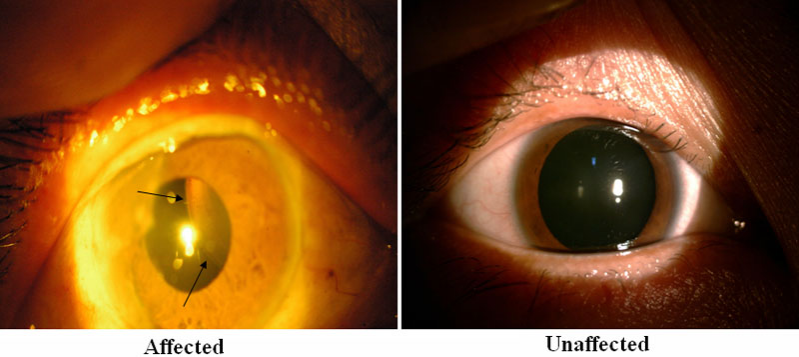
Slit lamp photograph showing dislocation of the patient’s lens (III:14 in Figure 1). Arrows indicate the edge of the lens.

We identified a five-generation family with sixteen confirmed individuals affected with isolated EL (Figure 2). Eleven patients’ blood samples were collected. All participated patients have received surgery. The onset age of patients with EL was around 15 to 30 years. Patient II-1 was a seventy-eight-year old woman who, except for the EL, had no other Mafan-related disorders. The stature of all the patients in this family was shorter than 1.70 m. We did not find the cardiovascular system, the skeleton system, and other Marfan-related syndromes in the collected ten patients.

### Linkage and haplotype analysis

We obtained positive logarithm-of-odds (LOD) scores with markers at 15q (Table 1). A maximum positive LOD score of 2.61 at θ=0.00 was obtained by marker D15S126. The haplotype shows complete cosegregation in all the 9 affected individuals who were studied (Figure 1).

**Table 1:** Result of linkage analysis.

** **	** **	**LOD scores at θ=**					
**Marker**	**Physical distance (M)**	**0.00**	**0.01**	**0.10**	**0.20**	**0.30**	**0.40**
D15S992	48.8	2.23	2.19	1.82	1.38	0.89	0.37
MTS-1	48.9	0.12	0.12	0.10	0.08	0.05	0.03
*FBN1*	48.7–48.9	** **	** **	** **	** **	** **	** **
D15S1082	49.0	2.06	2.02	1.67	1.26	0.80	0.33
D15S126	49.3	2.61	2.57	2.16	1.66	1.10	0.48

### Mutation analysis

**Figure 3:**
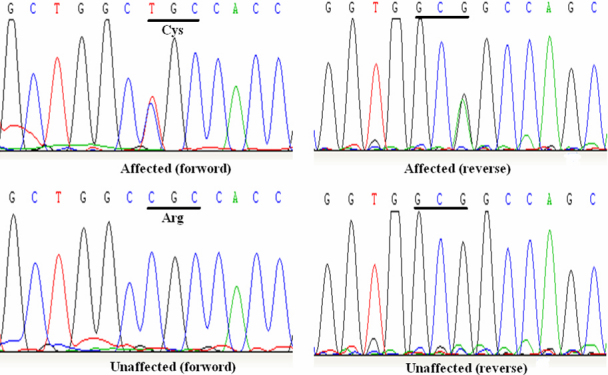
DNA sequence analysis of *FBN1* in unaffected and affected individuals. A heterozygous change C>T at codon 974 (CGC-C/TGC), resulting in the substitution of arginine to cysteine (p.R974C) in the affected individuals.

**Figure 4:**
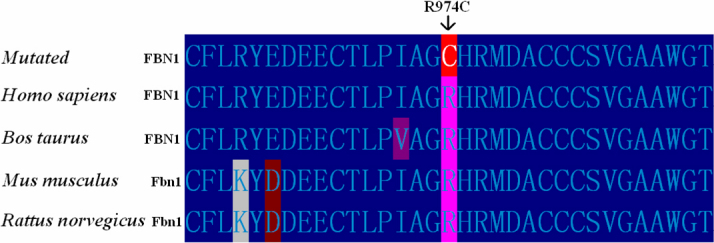
The alignment of amino acids of FBN1 from *Bos taurus*, *Rattus norvegicus*, *Mus musculus*, and *Homo sapiens*. The Arg974 residue is highly conserved during evolution.

Sequencing of *FBN1* shows a heterozygous C>T change in the affected individuals at nucleotide position c.2920 (Figure 3). This nucleotide change results in a cysteine substitution for a highly conserved arginine at codon 974 (p.R974C; Figure 4). All affected family members presented this nucleotide change. Haplotyping shows individual IV-15 was affected, while individual V-1 was unaffected. Mutation detection is consistent with haplotyping of those two members. This nucleotide substitution was not observed in 50 control subjects from the same ethnic background.

## Discussion

In the present study, we identified a novel mutation (R974C) in *FBN1* from a Chinese family with isolated EL. This missense variation is found in all the affected family members. The mutation leads to a cysteine substitution for highly conserved arginine in the 8-Cys repeat latent transforming growth factor-beta binding protein (LTBP) motif encoded by *FBN1* exon 25.

To date, over 600 mutations in *FBN1* have been reported. The relationship between the genotype and the phenotype is still not clear [12]. Some information is concluded from reported materials by researchers [13]. For instance, mutations in exons 24–32 of *FBN1* are usually associated with a severe form of Marfan syndrome (MFS), called neonatal MFS [13]. Mutations of cysteine substitution are usually associated with isolated EL [14]. On the another hand, some facts obstruct the conclusion, including mutations in the exons 24–32 of *FBN1* caused MFS and isolated EL, and the same mutation can cause MFS and isolated EL in different families. Since these conclusions were obtained from the clinical materials, but not from the pathogenic mechanism, we can not correlate the genotype and the phenotype from clinical symptoms only. So, we need to explain the pathogenic mechanism on the molecular biologic level.

Fibrillin-1 is a large (320 kDa) multidomain glycoprotein that is a main component of the 10 to 12 nm sized extracellular microfibrils found in a wide range of tissues. Fibrillin-1 is considered to be important for elastogenesis, elasticity, and homeostasis of elastic fibers [15,16].

In the previous experiments, researchers found that fibrillin-1 was not only a structural component of extracellular matrix but also was a mediator of the transforming growth factor-beta (TGFβ) signaling pathway [17,18]. Mutations that influence the TGFβ signaling pathway are believed to cause severe multiple defects [19].

Through a literature search, eighteen mutations of *FBN1* causing EL were found [8-10,14,20-23]. In these mutations, three mutations of *FBN1* can cause EL and MFS in different families. Mutations of cysteine substitution were found in fifteen families [8-10].

In this study, we found the extra created cysteine in the LTBP motif only results in isolated EL. This suggests that the correct cysteine localization and disulfide binding play an important role in the structural integrity of the lens suspensory ligaments. The change has no or slight effect in the extracellular matrix of other organs.

It is reported that some specified mutation can cause MFS and isolated EL in different families. This may be influenced by genetic or environmental modifiers.

In summary, we report a novel conserved arginine to cysteine substitution in exon 25 of *FBN1* which is associated with isolated EL. Our results further expanded the mutation spectrum of *FBN1* and provided useful materials to the further researches.

## Appendix 1. Table 1. *FBNI* primers and conditions for PCR.

To access the data, click or select the words “Appendix 1.” This will initiate the download of a compressed (pdf) archive that contains the file.
